# Limitations of transthoracic echocardiography in equine patients

**DOI:** 10.1002/vms3.906

**Published:** 2023-03-30

**Authors:** Valentina Vitale, Malene Laurberg, Gaby van Galen, Gunther van Loon

**Affiliations:** ^1^ Department of veterinary medicine CEU Cardenal Herrera ‐ University of Valencia Valencia Spain; ^2^ HS Hestepraksis Tarm Denmark; ^3^ Department of Veterinary Sciences University of Sydney Sydney Australia; ^4^ Private hospital Goulburn Valley Equine Hospital Congupna Australia; ^5^ Equine Cardioteam, Department of Large Animal Internal Medicine Ghent University Merelbeke Belgium

1

Transthoracic echocardiography (TTE) is part of every comprehensive cardiac examination in horses (Schwarzwald, 2019). However, the echocardiographic assessment of cardiac structures, chamber dimensions, and myocardial function is challenging and limited by a variety of technical, anatomic, and physiological factors that need to be considered (Vitale et al., 2021). More recently, transesophageal and three‐dimensional echocardiography have been described to overcome some of the limitations of the two‐dimensional traditional echocardiography, nevertheless they are not widely available in equine hospitals (Schwarzwald, 2018). During the ultrasonographic examination, the valves are inspected for the number and morphology of the leaflets and cusps, the integrity of the support apparatus and motion during the cardiac cycle (Marr, 2019). In the recently published article (Vitale et al., 2021), the authors described a 4‐year‐old Thoroughbred with mild aortic regurgitation suspected to be due to a quadricuspid aortic valve, a form of aortic valve dysplasia. Valvular dysplasia refers to a valve that is malformed, which may manifest as stenosis, insufficiency, or both (Scansen, 2019). The case presented in the article was referred to the hospital because of the development of paroxysmal atrial fibrillation, and on interrogation with colour flow Doppler, mild aortic and pulmonary regurgitation was identified. These mild regurgitations are unlikely to affect performance or to result in any clinical signs (Reef et al., 2014). Nevertheless, as described in the article, the authors repeatedly obtained images of the aortic valve, which were thought to be abnormal. This clinical commentary arises from the same authors because deeper examination of the recorded images and video loops of that echocardiography, and together with the opinion of a clinician with more experience in cardiology (GvL), indicated that this interpretation was most likely wrong. Indeed, while reviewing the recordings, in diastole, with the valve completely closed, it was possible to obtain static images that are in fact incompatible with a quadricuspid aortic valve (Figure 1). Although at the time of the article submission and publication, the authors genuinely believed that there was a likelihood of four cusps, with this clinical commentary they provide the readers with an update. Because static images are difficult to interpret, video loops are included to better evaluate the case presented (Videos S1 and S2). As already mentioned in the original paper (Vitale et al., 2021), when dealing with concave/convex structures in rapid movement, such as the cardiac valves, a slight off‐angle section plane can produce an artefactual image resembling more subdivided cusps (Figures 2 and 3). For the aortic valve, this typically occurs in diastole on a short axis view that is taken slightly too ventrally. On such a view, one can easily be misled by the images obtained. This error further supports the conclusion of the article regarding the difficulties and risks of misdiagnosis that transthoracic echocardiography includes (Malik et al., 2017; Orde et al., 2017). The lessons learned and the advice to other clinicians are as follows: 1) to be extremely careful in the evaluation of potentially abnormal structures during echocardiography; 2) to obtain contemporaneous electrocardiographic recordings with the ultrasound machine in order to correctly identify the timepoint within the cardiac cycle; and 3) to re‐evaluate stored video loops offline and in slow motion in case of any doubts and/or perform advanced cardiac imaging (3D or intracardiac ultrasound).

**FIGURE 1:**
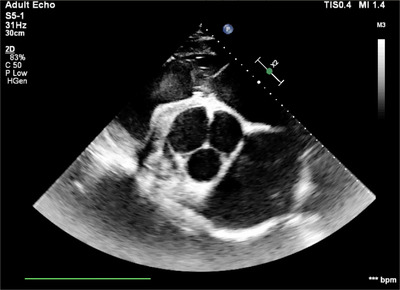
B‐mode right parasternal short‐axis left ventricular outflow tract view in diastole showing the three cusps of the aortic valve

**FIGURE 2:**
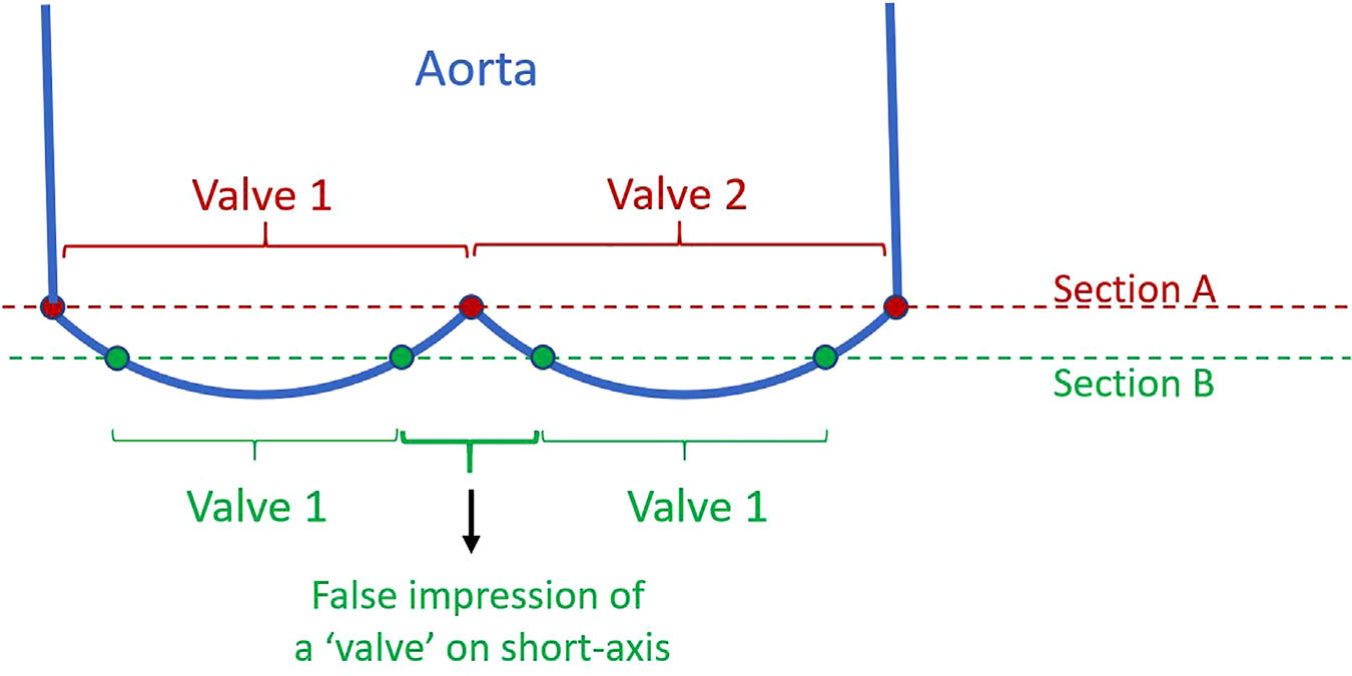
Schematic drawing of a long‐axis view of a hypothetical valve (with two cusps for ease), explaining the effect of a slightly altered section plane through that valve. Section A (red dotted line) is taken at the level where both cusp edges touch each other and would produce a correct cross‐section displaying both leaflets. Section B (green dotted line) is taken through the curved part of the valve, away from closure level. This creates four section points through the valve instead of three, giving the false impression on the short‐axis image that an additional valve is present

**FIGURE 3:**
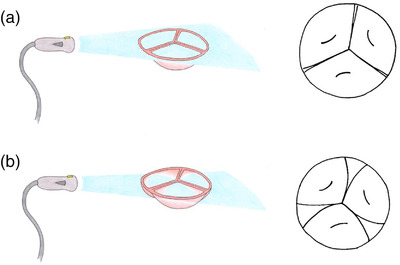
Schematic drawing of the ultrasound beam transecting the aortic valve in short axis (left) and the corresponding image (right). In (a), the beam transects at the level of valve closure, producing an image with three cusps. In (b), the beam transects just ventral to the level of valve closure, giving the false impression of additional valves

## AUTHOR CONTRIBUTIONS

*Writing–original draft*: Valentina Vitale. *Investigation*: Malene Laurberg. *Writing–review and editing*: Gaby Van Galen. *Project administration and writing–review and editing*: Gunther van Loon.

## ETHICS STATEMENT

No ethical statement has been uploaded as no animals have been used in this commentary article.

2

### PEER REVIEW

The peer review history for this article is available at https://publons.com/publon/10.1002/vms3.906.

## Supporting information

VIDEO 1 B‐mode right parasternal short‐axis view of the aortic valve. In early diastole, the ultrasound beam transects at the level of valve closure, showing 3 cusps. During the remainder of diastole, the transection is located just ventral to valve closure, giving the false impression of an additional aortic cusp

VIDEO 2 B‐mode left parasternal short‐axis view of the aortic valve giving the false impression of an additional aortic cusp.

## Data Availability

Data sharing not applicable to this article as no datasets were generated or analysed during the current study.
